# The Tight Junction Associated Signalling Proteins ZO-1 and ZONAB Regulate Retinal Pigment Epithelium Homeostasis in Mice

**DOI:** 10.1371/journal.pone.0015730

**Published:** 2010-12-30

**Authors:** Anastasios Georgiadis, Marion Tschernutter, James W. B. Bainbridge, Kamaljit S. Balaggan, Freya Mowat, Emma L. West, Peter M. G. Munro, Adrian J. Thrasher, Karl Matter, Maria S. Balda, Robin R. Ali

**Affiliations:** 1 Department of Genetics, UCL Institute of Ophthalmology, University College London, London, United Kingdom; 2 Department of Cell Biology, UCL Institute of Ophthalmology, University College London, London, United Kingdom; 3 Electron Microscopy Unit, UCL Institute of Ophthalmology, University College London, London, United Kingdom; 4 Molecular Immunology Unit, UCL Institute of Child Health, University College London, London, United Kingdom; University of Colorado, Boulder, United States of America

## Abstract

Cell-cell adhesion regulates the development and function of epithelia by providing mechanical support and by guiding cell proliferation and differentiation. The tight junction (TJ) protein zonula occludens (ZO)-1 regulates cell proliferation and gene expression by inhibiting the activity of the Y-box transcription factor ZONAB in cultured epithelial cells. We investigated the role of this TJ-associated signalling pathway in the retinal pigment epithelium (RPE) *in vivo* by lentivirally-mediated overexpression of ZONAB, and knockdown of its cellular inhibitor ZO-1. Both overexpression of ZONAB or knockdown of ZO-1 resulted in increased RPE proliferation, and induced ultrastructural changes of an epithelial-mesenchymal transition (EMT)-like phenotype. Electron microscopy analysis revealed that transduced RPE monolayers were disorganised with increased pyknosis and monolayer breaks, correlating with increased expression of several EMT markers. Moreover, fluorescein angiography analysis demonstrated that the increased proliferation and EMT-like phenotype induced by overexpression of ZONAB or downregulation of ZO-1 resulted in RPE dysfunction. These findings demonstrate that ZO-1 and ZONAB are critical for differentiation and homeostasis of the RPE monolayer and may be involved in RPE disorders such as proliferative vitroretinopathy and atrophic age-related macular degeneration.

## Introduction

Retinal function is dependent on the retinal pigment epithelium (RPE), which is a monolayer of tightly connected pigmented cells underlying the photoreceptor cell layer. RPE cells not only support the function of photoreceptors, they also form the outer blood-retinal barrier (BRB) that prevents fluid from choroidal vessels from entering the retina [1], [2]. Breakdown of the BRB can lead to visual loss in a number of ocular disorders. However, the molecular mechanisms underlying RPE homeostasis are not completely understood.

Cell-cell adhesion plays a key role in epithelial cell function and several junctional components are dual localisation proteins, called NACos (Nucleus and Adhesion Complexes proteins), that play a role in signalling to the nucleus, cell proliferation and differentiation [3]. Tight junctions (TJs) are a type of cell-cell adhesion that have a fundamental role for the BRB function because they regulate paracellular diffusion across epithelia [4]. They also separate apical and lateral membrane components, and take part in signalling pathways involved in epithelial proliferation, gene expression and differentiation [5], [6]. ZO-1 is a membrane-associated TJ adaptor protein that links junctional membrane proteins to the cytoskeleton and signalling plaque proteins [7]. ZONAB (ZO-1-associated nucleic-acid-binding protein) is a Y-box transcription factor that binds to the SH3 domain of ZO-1. Binding of ZONAB to ZO-1 results in cytoplasmic sequestration and, hence, inhibition of ZONAB transcriptional activity [5], [6]. ZONAB interacts with the cell cycle kinase cdk4 and regulates the transcription of cell cycle genes such as cyclin D1 and PCNA, providing a molecular explanation for the role of ZO-1/ZONAB pathway in regulating proliferation of epithelia cells in culture [8], [9], [10]. Little is known about the role of ZO-1 and ZONAB *in vivo*. Nuclear translocation of ZONAB correlates with increased proliferation in the colonic epithelium of ethanol-fed mice and in adenomas of chronic alcoholics, suggesting a possible involvement in alcohol-induced gastrointestinal transformation [11]. ZONAB also seems to negatively regulate goblet cell differentiation, acting by suppressing AML1 and KLF4 [12]. During mouse kidney ontogeny, ZONAB expression decreased and inversely correlated with increasing apical differentiation, reflected by maturation of the brush border and extension of the primary cilium [13]. Thus, these studies suggest that decreased ZONAB expression correlates with differentiation. However, the effect of ZONAB overexpression on differentiation has not been shown yet *in vivo*.

During the last decade, numerous studies have demonstrated that the eye – and in particular the RPE – provides a valuable model system for the evaluation of the effects of gene transfer using viral vectors due to its easy accessibility [14], [15], [16]. Here, we describe the use of HIV-based lentiviral vectors to manipulate the expression of junctional signalling molecules in mouse RPE *in vivo*. Our results demonstrate that lentiviral vectors are efficient tools to regulate junctional proteins *in vivo* and indicate that ZO-1 and ZONAB are important for RPE homeostasis as their deregulation leads to changes in cell proliferation and morphology features of epithelial-mesenchymal transition *in vivo*.

## Materials and Methods

### Constructs and vector production

The targeting construct for ZO-1 was created by sub-cloning the target hairpins into the mU6pro plasmid and subsequent cloning into the lentiviral pHR-SIN backbone as previously described [10]. The resulting vector was named LNT.shZO-1. The sense strand of the targeting hairpins was 5′-AAGATAGTTTGGCAGCAAGAG-3′ for ZO-1. The LNT.shGFP vector that targets humanised renilla green fluorescent protein (hrGFP) expression was used as a control. Its sense strand of the GFP-targeting hairpin was 5′-GTTCATCTGCACCACCGGCAAGT-3′. Lentiviral vectors expressing either ZONAB or hrGFP were generated by using the Gateway® Cloning Kit (Invitrogen). The cDNA was cloned between the LTRs of the lentiviral backbone downstream of the ubiquitous active spleen focus-forming virus (SFFV) promoter. The resulting vectors were named LNT.ZONAB and LNT.hrGFP. Lentiviral production was carried out as previously described [14].

### Subretinal injections and angiography

Six to eight week old female wild-type C57BL/6 mice were used for this study (n = 40). All animals were cared for in accordance with the UK Home Office license (PPL 70/6956) with approval from the Institute of Ophthalmology ethics committee. Mice were anaesthetised by intraperitoneal injections of Dormitor (1 mg/ml, Pfizer Pharmaceuticals, UK) and ketamine (100 mg/ml, Fort Dodge Animal Health, UK) mixed with sterile water in the ratio 5∶3∶42. Surgery was performed under direct retinoscopy through an operating microscope as described elsewhere [17]. Two µl of virus suspension were injected to produce a bullous retinal detachment in the superior and inferior hemisphere of each eye. Where appropriate, 0.2 ml of a 100 ng/ml 5-bromo-2-deoxyuridine solution (BrdU; Sigma, UK) was injected intraperitoneally following the subretinal vector administration and injections were repeated daily for 5 days. For fluorescein angiography, 0.2 ml of 2% fluorescein sodium diluted in water was administered by intraperitoneal injection five minutes after the induction of anaesthesia. A Kowa Genesis small animal fundus camera equipped with appropriate excitation and barrier filters was used to obtain fluorescein angiograms at early (90 s after fluorescein injection) and late (7 min) phases of dye transit. At the early phase, the retinal vasculature is clearly defined by the intravascular fluorescein dye. At the late phase, any extravascular leakage or RPE loss is evident as patches of topical hyperfluorescence. Both the superior and inferior hemispheres were individually photographed in rapid succession (within 15 s). The contralateral eye was then immediately photographed.

### Semithin, ultrathin and cryosections

Mice were sacrificed at various time points and the eyes were immediately orientated with a nasal stitch. The eyes were fixed in 3% glutaraldehyde and 1% paraformaldehyde buffered to pH 7.4 with 0.07 M sodium cacodylate-HCl buffer, the cornea and lens removed and the eye cups were processed as previously described [16]. Semithin sections (0.7 µm) were cut using a Leica ultracut S microtome fitted with a diamond knife (Diatome histoknife). Sections were stained with 1% toluidine blue stain and slides were mounted with DPX after the sections had dried. Ultrathin sections (70 nm) were cut using a Leica ultracut S microtome fitted with a diamond knife for ultrathin sections (Diatome histoknife for ultrathin sections). Sections were taken of treated areas of retinae and collected onto grids. Sections were stained with uranyl acetate for 10 min and lead citrate for 7 min and then washed with dH_2_O. After the sections had dried they were analysed by electron microscopy (JEOL 1010 TEM). For cryosections, eyes were retrieved and immediately immersed in 4% paraformaldehyde for 2 hr. After fixation the eyes were embedded and frozen in optimum cutting temperature medium and 12 µm thick sections were cut using a Bright cryostat.

Quantification of RPE features for each treatment group was carried out on 20 retinal cryosections from 4 eyes (5 sections from each eye). The presence of two features was assessed in each section: RPE pyknosis and RPE breaks. A section was scored positive for a feature if it occurred once or more on that section. Thus, for each feature the maximum possible score was 20. The percentage of sections scored positive for each feature was plotted. Error bars indicate standard deviation of the mean between different eyes.

### Laser capture microdissection and RT-PCR

Cryosections (20 µm thick) from treated eyes (n = 4) were collected on PEN-membrane coated slides NF 1.0 (ZEISS ltd., UK) and RPE cells were immediately collected using a PALM Robomover Axiovert 200 miscroscope (ZEISS ltd., UK). Approximately 100 RPE cells were laser-cut and catapulted into a silicon-embeded AdhesiveCap eppendorf (ZEISS ltd., UK). Cells were lysed immediately and total RNA extraction was performed using the RNeasy MicroKit (Qiagen ltd., UK). Reverse transcription on total mRNA lysates was performed using the QuantiTect Whole Transcriptome Amplification kit (Qiagen ltd., UK) following the manufacturer's directions.

PCR reactions to assess EMT marker expression were performed on amplified cDNAs using the following primers: GFAP (glial fibrillary acidic protein) forward: 5′-ACAGACTTTCTCCAACCTCCAG- 3′. GFAP reverse: 5′-CCTTCTGACACGGATTGGT-3′. Vimentin forward: 5′-TGCGAGAGAAATTGCAGGA-3′. Vimentin reverse: 5′-GTGCCAGAGAAGCATTGTCA-3′. N-cad (N-Cadherin) forward: 5′-CCTCCATGTGCCGGATAG-3′. N-cad reverse: 5′-CACCAGAAGCCTCCACAGAC-3′. cD1 (cyclin D1) forward: 5′-GAGATTGTGCCATCCATGC-3′. cD1 reverse: 5′-CTCCTCTTCGCACTTCTGCT-3′. Snail1 forward: 5′-GTCTGCACGACCTGTGGAA-3′. Snail1 reverse: 5′-CAGGAGAATGGCTTCTCACC-3′. CtBP1 (C-terminal binding protein 1) forward: 5′-CCAGGATCCGTGGAGAGAC-3′. CtBP1 reverse: 5′-GGACGTTGAAGCCAAAAGC-3′. α-SMA (alpha smooth muscle actin) forward: 5′-CAACCGGGAGAAAATGACC-3′. α-SMA reverse: 5′-CAGTTGTACGTCCAGAGGCATA-3′. Cyclic conditions were: 94°C for 30 seconds, 60°C for 1 min, repeated for 40 cycles.

### Immunohistochemistry

Cryosections were air-dried for 10 min and blocked for 1 hr with a TBS-T solution containing 3% BSA, Triton-X (0.5%), and 5% serum of the species the 2° antibody was raised in. The 1° antibodies directed against either ZO-1 (rabbit polyclonal, 4913, 1/200 dilution [8]), ZONAB (rabbit polyclonal, 5599, 1/500 dilution [8]), RPE65 (mouse monoclonal, Chemicon, UK, 1/500 dilution) or BrdU (rat monoclonal, Abcam, UK, 1/1000 dilution) were then added and sections were incubated overnight at 4°C. After washing with TBS-T, sections were incubated with the respective 2° antibody in blocking solution (1/500 dilution) for 2 hr at room temperature. The sections were counterstained with Hoechst 33342 (Sigma, UK) or propidium iodide (PI; Sigma, UK) and mounted on mounting medium (DAKO). For BrdU immunostaining, sections were pre-treated with 2 M HCl for 30 min at 37°C prior to the addition of the 1° antibody. The slides were analysed using the 3-laser ZEISS LSM 510UV Confocal Imager. Alternatively, sections were treated for haematoxylin and eosin staining before capturing images with a Leica DC 500 digital camera mounted on the microscope.

Quantification of BrdU positive cells was carried out on 20 retinal cryosections from 4 eyes per treatment group. Total RPE cells were first counted per section (average total number of RPE cells/section = 160) and each BrdU positive RPE cell was subsequently counted. The percentage values account for the average number of BrdU cells per treatment group towards the average total number of RPE cells counted. Error bars indicate standard deviation of the mean.

## Results

### *In vivo* RPE transduction

HIV-based lentiviral vectors can be used to mediate efficient gene delivery specifically to the RPE [14]. We therefore generated HIV-1–based vectors to manipulate the expression of the tight junction associated proteins ZO-1 and ZONAB. ShRNAs targeting ZO-1, or hrGFP were driven by a U6 RNA polymerase III promoter and were based on sequences previously used to downregulate ZO-1 in cultured cells [10]. Vectors for ZONAB and hrGFP overexpression used a spleen-focus forming virus (SFFV) promoter to drive expression of the respective cDNAs.

**Figure 1:**
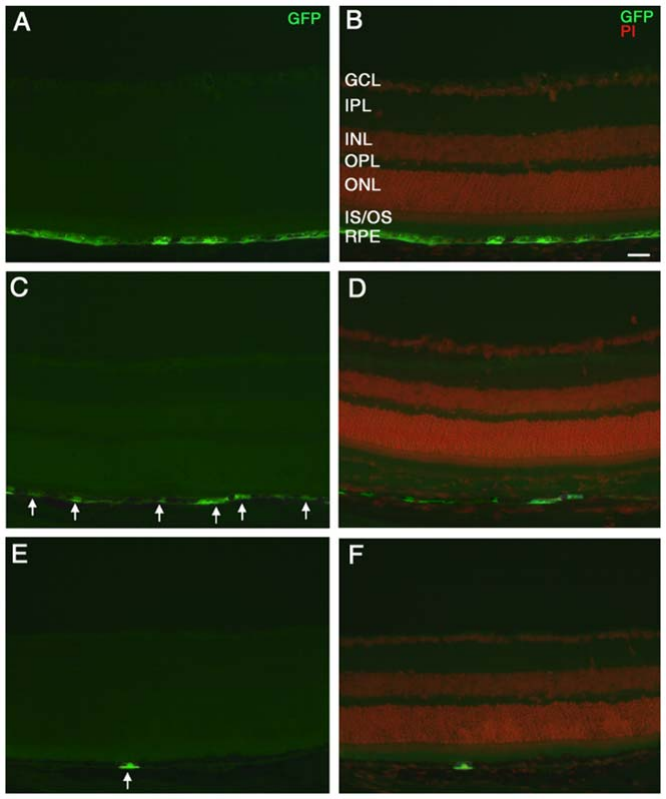
RPE transduction following subretinal delivery of LNT.hrGFP. Retinal cryosections were obtained from eyes 14 days after subretinal injection of LNT.hrGFP at titres of 10^8^ T.U./ml (**A, B**), 10^7^ T.U./ml (**C, D**) and 10^6^ T.U./ml (**E, F**). Expression of GFP (green) was restricted to the RPE (left panel). Propidium iodide (red) was used as a nuclear counterstain (right panel, merged with GFP). White arrows, GFP-positive cells. GCL, ganglion cell layer; IPL, inner plexiform layer; INL, inner nuclear layer; OPL, outer plexiform layer; ONL, outer nuclear layer; IS, inner segments; OS, outer segments; RPE, retinal pigment epithelium; Size bar, 20 µm. n = 4 per treatment group.

We first assessed transduction levels using serial dilutions of LNT.hrGFP. Wild-type (wt) mice (n = 12) were subretinally injected and transgene expression within the treated area was analysed two weeks post injection (p.i.). Transduction of the entire RPE monolayer was observed following injection of a titre of 10^8^ transducing units/ml (T.U./ml) (Fig. 1A, B). Injection of a titre of 10^7^ T.U./ml resulted in discontinuous transduction of the RPE monolayer (Fig. 1C, D). At 10^6^ T.U./ml, minimal RPE transduction was observed with expression of hrGFP by the occasional RPE cell (Fig. 1E, F). No GFP expression was evident after injection of vector at a titre of 10^5^ T.U./ml (data not shown). Even at the highest titre, GFP expression was only observed in RPE cells, supporting the specificity of the viral vector [14]. Similar levels of GFP expression as well as RPE specificity were observed at 5, 10, 30 and 60 days post injection (data not shown).

In order to assess the role of ZO-1 and ZONAB in RPE cells we used lentiviral vectors expressing ZONAB or shRNAs (LNT.shZO-1and LNT.shGFP) at two titres. A titre of 10^7^ T.U./ml was effective in altering protein expression and such manipulations correlated with changes in cell proliferation. A titre of 10^8^ T.U./ml resulted in a more severe phenotype. Prior to the transduction experiments described below, preliminary experiments were conducted in order to identify the most appropriate time points for detailed analysis. We observed knockdown and induction of proliferation after 5 days whereas retinal degeneration was apparant at later time points (at least 10 days post injection).

### Manipulation of ZO-1 or ZONAB levels in RPE *in vivo*

**Figure 2:**
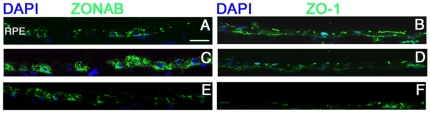
Lentivirally-mediated modulation of ZO-1 and ZONAB expression. Retinal cryosections were obtained from eyes 5 days after the subretinal injections of LNT.shGFP (**A,B**), LNT.ZONAB (**C,D**) or LNT.shZO-1 (**E,F**) at 10^7^ T.U./ml. Immunostaining was performed using antibodies against ZONAB (left panel) and ZO-1 (right panel). Following injection of LNT.shGFP, ZONAB can only be detected at low levels in the RPE (**A**) and ZO-1 was observed in the RPE as apical dots depending on the section (**B**). Subretinal injection of LNT.ZONAB resulted in an elevation of ZONAB levels in RPE cells (**C**) and did not affect ZO-1 levels (**D**). Depletion of ZO-1 expression resulted in a slight increase in ZONAB expression (**E**) and decreased of ZO-1 expression (**F**) compared with control eyes (**B**). Nuclei were counterstained with DAPI. White arrows, RPE monolayer. Size bar, 20 µm. n = 4 per treatment group.

We first tested the ability of the lentiviral vectors to alter the levels of ZO-1 and ZONAB. Following injection of the control LNT.shGFP virus *in vivo*, basal levels of ZONAB in RPE cells and ZO-1 immunofluorescence at cell-cell junctions were observed (Fig. 2A,B). Increased levels of ZONAB could be observed following subretinal injection of LNT.ZONAB confirming the integrity of the expression cassette (Fig. 2C). No change in ZO-1 immunostaining was observed after injection of LNT.ZONAB (Fig. 2D), whilst a marked reduction was observed 5 days after subretinal injection of LNT.shZO-1 (Fig. 2F). Unexpectedly, slightly increased fluorescence of endogenous ZONAB staining was observed in the LNT.shZO-1 treated eyes (Fig. 2E). These results demonstrate that manipulation of ZONAB or ZO-1 levels in RPE cells *in vivo* can be obtained using HIV-1-based lentiviral vectors.

### ZO-1 downregulation or ZONAB overexpression results in increased RPE cell proliferation *in vivo*

**Figure 3:**
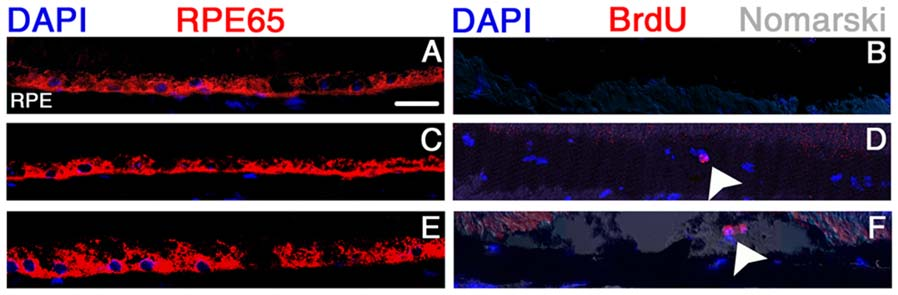
Manipulation of ZO-1 and ZONAB expression increases RPE cell proliferation. Retinal cryosections were obtained from eyes 5 days after the subretinal injections of LNT.shGFP (**A,B**), LNT.ZONAB (**C,D**) or LNT.shZO-1 (**E,F**) at 10^7^ T.U./ml. BrdU was injected intraperitoneally following the subretinal vector administration. Immunostaining was performed using antibodies against RPE65 (left panel) and BrdU (right panel). In eyes injected with LNT.shGFP, high levels of RPE65 were evident in RPE cells (**A**) and there was no evidence of proliferation judged by the absence of BrdU staining (**B**). Overexpression of ZONAB did not change RPE65 levels (**C**) but increased the number of BrdU positive cells, indicative of proliferation (**D**, white arrowhead). Delivery of LNT.shZO-1 did not affect RPE65 expression (**E**) but increased BrdU positive cells suggesting RPE proliferation (**F,** white arrowhead). Nuclei were counterstained with DAPI. Size bar, 20 µm. n = 4 per treatment group.

ZO-1/ZONAB signalling controls G_1_/S phase transition and differentiation of epithelial cells in culture [9], [10]. We therefore analysed RPE differentiation by testing the expression of an RPE-specific marker, RPE65, five days after injection of LNT.shGFP, LNT.ZONAB or LNT.shZO-1 [18]. Immunostaining of RPE65 in the RPE following injection of LNT.shGFP, LNT.ZONAB or LNT.shZO-1 was not altered (Figure 3) indicating that after five days transduced RPE cells retained their epithelial character.

**Figure 4:**
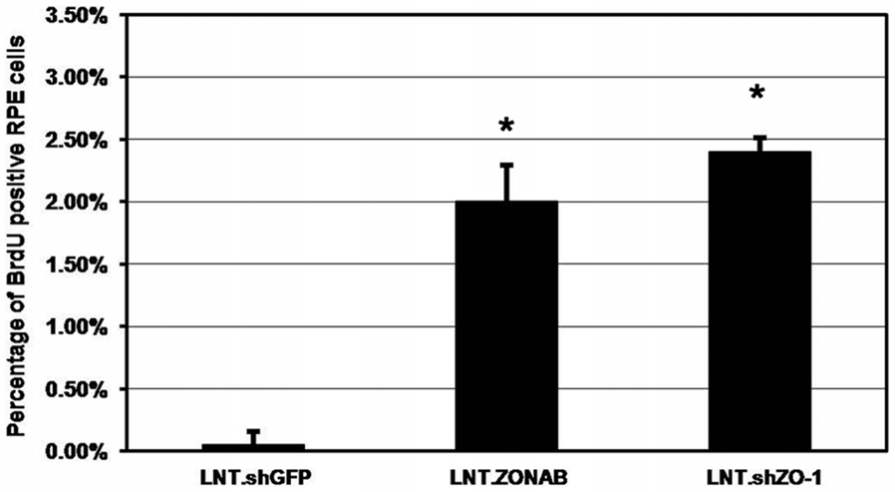
Quantification of BrdU positive RPE cells. BrdU positive RPE cells and total RPE cell numbers were counted in the middle of the treated area of retinal cryosections obtained from subretinally injected mice 5 days after vector administration at a titre of 10^7^ T.U./ml. Following injection of LNT.shZO-1 or LNT.ZONAB BrdU positive cells increased to 2.4% and 2.0% of total RPE cell number, respectively, whereas very few BrdU positive cells were identified in LNT.shGFP (0.05%) treated eyes. (*P<0.001 compared with LNT.shGFP control. Student's *t*-test. n = 4 [20 measurements from 4 eyes per treatment group]).

In epithelial cell lines, ZONAB regulates proliferation by stimulating G1/S phase progression while the postnatal RPE is not proliferative [19]. We therefore tested whether manipulation of ZONAB and ZO-1 expression in RPE *in vivo* affects cell cycle entry using BrdU incorporation. Whereas injection of control virus did not induce proliferation (Fig. 3B), proliferating cells were detected in eyes following injection of either LNT.ZONAB (Fig. 3D) or LNT.shZO-1 (Fig. 3F). We also quantified the number of BrdU-positive cells following injection of the lentiviral vectors (Fig. 4), confirming the induction of proliferation by downregulation of ZO-1 and overexpression of ZONAB. Thus, treatments that result in increased ZONAB activity, either by direct overexpression or by downregulation of its inhibitor, stimulate proliferation in RPE cells *in vivo*.

### ZO-1 and ZONAB regulate RPE cell morphology and differentiation *in vivo*

**Figure 5:**
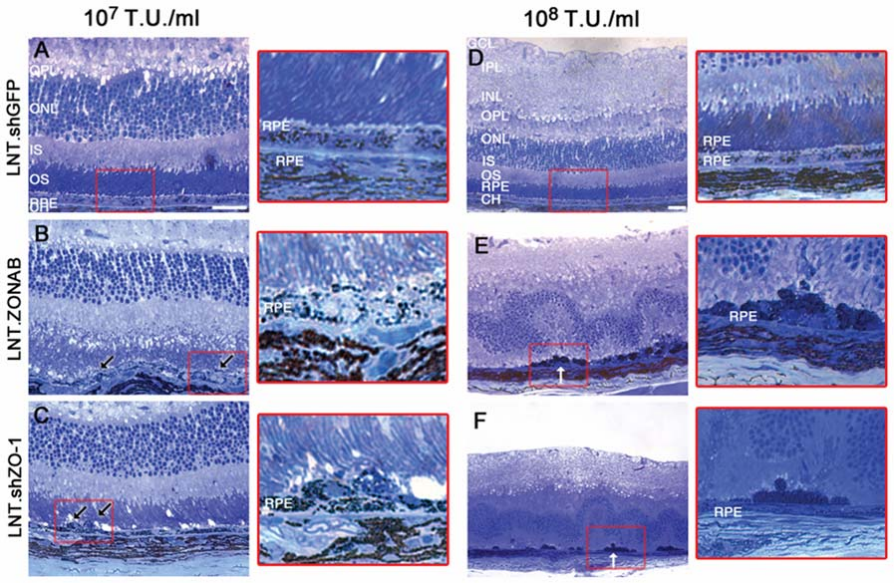
Downregulation of ZO-1 or overexpression of ZONAB affects retinal morphology. Retinal semithin sections were obtained after 10 days of subretinal injection of LNT.shGFP (**A,D**), LNT.ZONAB (**B,E**) or LNT.shZO-1 (**C,F**) at 10^7^ T.U./ml (left panel, ×40 magnification) and 10^8^ T.U./ml (right panel, ×20 magnification). Areas in red rectangles are shown in higher magnification. The LNT.shGFP treated eyes (**A,D**) exhibit normal retinal architecture showing that there are no adverse effects induced either by the lentiviral vector itself or by the expression of shRNA. Following injection of either LNT.ZONAB (**B,E**) or LNT.shZO-1 (**C,F**) signs of RPE puknosis and multilayerisation (black arrows) as well as retinal folding and rosette formation in areas corresponding to those with severe RPE abnormalities (white arrows) were observed. GCL, ganglion cell layer. IPL, inner plexiform layer. INL, inner nuclear layer. OPL, outer plexiform layer. ONL, outer nuclear layer. IS, inner segments. OS, outer segments. RPE, retinal pigment epithelium. CH, choroid. Size bar, 20 µm. n = 4 per treatment group.

**Figure 6:**
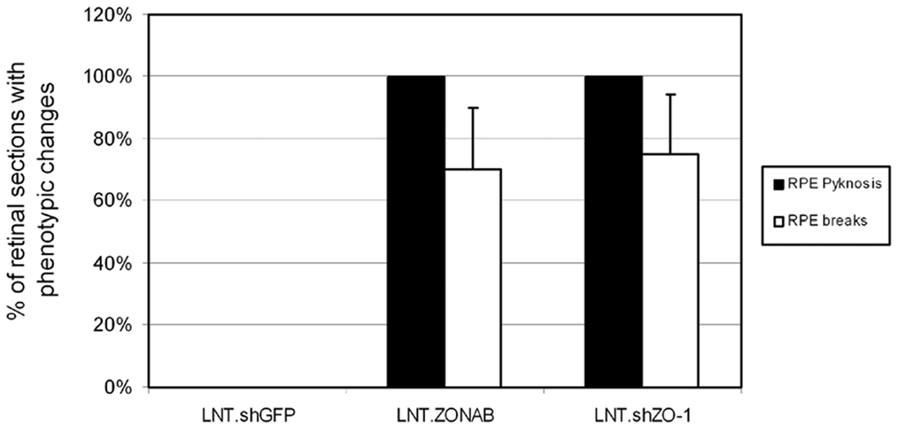
Downregulation of ZO-1 or overexpresion of ZONAB induces RPE pyknosis and breaks. Two features were assessed: RPE pyknosis and RPE breaks 10 days after subretinal injection of vectors at 10^8^ T.U./ml. Pyknotic RPE cells were defined as hyperpigmented cells that were not within a continuous monolayer. Percentage of retinal sections in which RPE pyknosis and breaks were observed is plotted. LNT.shZO-1 and LNT.ZONAB treated eyes contained many pyknotic RPE cells. RPE breaks or cell loss occurred adjacent to pyknotic RPE areas in LNT.shZO-1 or LNT.ZONAB treated eyes, respectively. (n = 4, 20 measurements from 4 eyes per treatment group).

**Figure 7:**
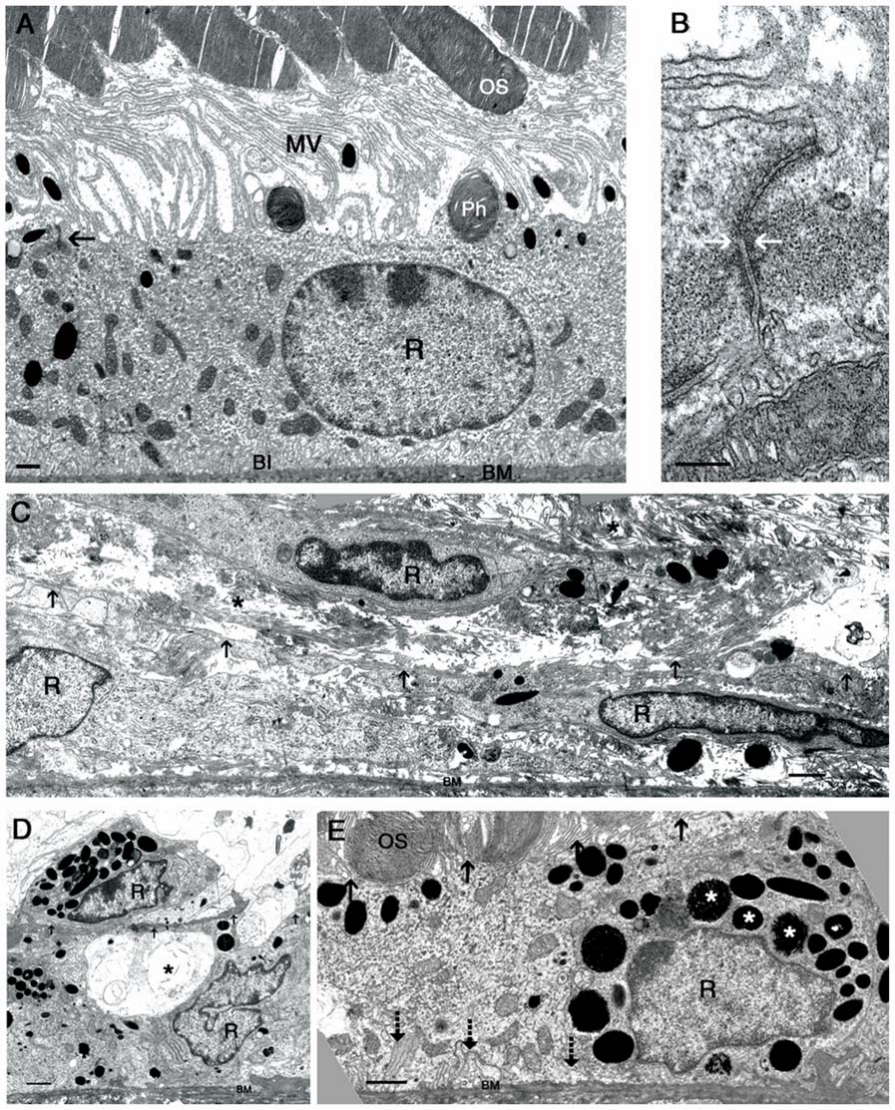
Ultrastructure of the RPE after 10 days following subretinal injection of vectors at 10^8^ T.U./ml. In LNT.shGFP treated eyes (**A**), the RPE monolayer lies on Bruch's membrane, has apical microvilli towards the photoreceptor outer segments the cells are interconnected by tight junctions (**A,** black arrow. **B**, white arrows). In eyes either with depleted levels of ZO-1 (**C**) or overexpressing ZONAB (**D**), the RPE monolayer was highly disorganised with a marked loss of the epithelial monolayer characteristics, areas of RPE cells located on top of each other (see Figure 6: RPE pyknosis) and accumulation of extracellular debris was also seen (**C,** asterisk). RPE cells appeared flattened and elongated with absent microvilli (black arrows indicate the flat apical side of the cell), reduced basal infoldings, and mesenchymal-like morphology. In addition, numerous vacuoles were present within the cells (**D,** asterisk). In areas adjacent to RPE breaks (**E**), the RPE retained some of its epithelial characteristics, such as, microvilli present on the apical membrane and intracellular basal infoldings. However, melanin vesicle maturation was defective (asterisks). R, RPE cell nuclei. OS, photoreceptor outer segments. Mv, microvilli. BM, Bruch's membrane. BI, basal infoldings. Ph, phagosome. Size bar, 1 µm (except in **B**, 200 nm). n = 4 per treatment group.

As manipulation of ZO-1 or ZONAB increases RPE cell proliferation (Fig. 3D, F and 4), we next analysed changes in RPE cell morphology and retinal integrity after manipulation of ZONAB or ZO-1 by subretinal injection of vectors at titres of 10^7^ and 10^8^ T.U./ml (Fig. 5). Semithin sections of treated eyes were analysed to determine morphological changes. Injection of control virus did not affect the morphology of the RPE and the neuroretina (Fig. 5A,D). Mild morphological changes were observed after 5 days of subretinal injection of LNT-shZO-1 or LNT-ZONAB vectors (Fig. 2 and 3). However, more pronounced morphological changes were observed after 10 days of subretinal injection of LNT-shZO-1 or LNT-ZONAB vectors (Fig. 5, 6 and 7). In general, a disrupted RPE monolayer and altered photoreceptor morphology were observed. We observed two characteristic structural changes of the RPE monolayers, such as pyknosis and breaks. Pyknosis was identified and quantified as hyperpigmented RPE cells that do not adhere to the monolayer conformation (Fig. 5E). Breaks were identified as discontinuities in the RPE monolayer (Fig. 5F). Figure 6 shows the quantification of such phenotypes. Photoreceptor outer segments appeared disorganised and with extracellular gaps (Fig. 5C). Accumulation of debris in the inter-retinal space indicated that the phagocytotic function of the RPE was compromised suggesting loss of normal RPE function. In some areas, the presence of macrophage-like pigmented cells on the apical surface of the RPE suggested the disruption of the posterior BRB and possible leukocyte infiltration (Fig. 5F). Thus, downregulation of ZO-1 or increase of ZONAB expression cause RPE dedifferentiation that leads to widespread degeneration of the retina after 10 days of subretinal injections of the respective vectors, suggesting a severe loss of RPE function.

RPE morphological changes were observed after delivery of either vector titre. However, following injection of vector at the higher titre of 10^8^ T.U./ml, retinal foldings and rosette formations were evident indicating an impact on the integrity of the neuroretina. These alterations were particularly evident in the outer nuclear layer (ONL), which consists of photoreceptor nuclei. Downregulation of ZO-1 or overexpression of ZONAB induced predominantly pyknotic cells that appeared to have lost polarity and formed cell aggregates compromising monolayer integrity. In some areas these cells invaded the inner photoreceptor matrix (IPM) (Fig. 5F). These pyknotic cells RPE breaks occurred in both treatment groups mostly adjacent to pyknotic RPE areas. Thus, the levels of ZO-1 and ZONAB expression are important determinants of RPE cell morphology and differentiation *in vivo*, indicating that ZO-1 and ZONAB are critical for RPE cells to maintain their differentiated phenotype and to fulfil their support function for the neural retina.

To evaluate the morphological changes observed in semithin sections in more detail, we used transmission electron microscopy (TEM) to assess the ultrastructural changes of the RPE. Normal RPE consists of a monolayer of tightly packed cells that exhibit a highly polarised epithelial phenotype. They are separated from the choroid by Bruch's membrane (BM) (Fig. 7A). Adjacent RPE cells are interconnected via a network of cell-cell intercellular junctions with tight junctions located towards the apical surface of the lateral membrane (Fig. 7A,B). The apical membranes of RPE cells are covered with finger-like microvilli that surround the photoreceptor outer segments. RPE cells with reduced expression of ZO-1 or increased expression of ZONAB were flatter and more elongated in comparison with the cuboidal architecture of normal RPE cells suggesting that they had lost polarisation (Fig. 7C, D). Basal infoldings were absent or highly disorganised and many cells did not adhere closely to the BM. Apical microvilli were also absent, disrupted cell-cell junctions and some multilayerisation were observed. The cells were often surrounded by cell debris or extracellular matrix components (Fig. 7C). At areas near a monolayer break point, the RPE was thinner and although the cells retained some of their normal morphological characteristics, the microvilli on the apical membrane and the basal infoldings appeared disorganised. Whereas only mature melanin vesicles can be observed in a healthy postnatal RPE, different stages of melanin vesicle maturation were observed in the cells near monolayer breaks (Fig. 7E, asterisks). Melanin vesicle maturation occurs prenatally and although some controversy remains over whether melanin synthesis occurs in the adult RPE, presence of immature melanin vesicles might be a sign of diseased or stressed RPE [20]. Thus, downregulation of ZO-1 or overexpression of ZONAB in RPE cells *in vivo* increase cell proliferation and trigger morphological changes.

**Figure 8:**
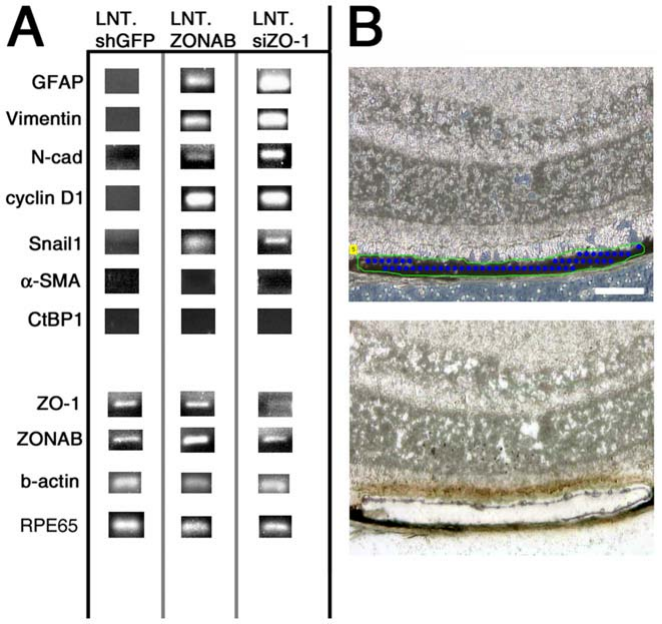
Downregulation of ZO-1 or overexpresion of ZONAB induces expression of EMT markers. RT-PCR amplification of mesenchymal markers (**A**) on RNA isolated from laser captured RPE cells (**B**) from eyes collected 10 days after subretinal injection with either LNT.shGFP, LNT.ZONAB or LNT.shZO-1. Specific primers for GFAP, vimentin, N-cadherin, cyclin D1, Snail1, CtBP1 and α-SMA were used as described in Materials and Methods. EMT markers associated with cell cycle progression such as Snail1 and cyclin D1 were found to be upregulated in either LNT.ZONAB or LNT.shZO-1 treated eyes. EMT morphological markers such as GFAP, vimentin and N-cadherin were also found to be upregulated in eyes showing overexpression of ZONAB or downregulation of ZO-1. (**B**) Indicative image of selected RPE cells before (upper image) and after (lower image) laser capture microdissection. Approximately 100 cells (three times the area indicated) were collected per eye per treatment group. Size bar, 20 µm. n = 4 per treatment group.

When epithelial cells undergo mesenchymal transition, they lose their epithelial character and acquire a fibroblastic phenotype and migratory properties [21], [22], [23], [24], [25], [26], [27], [28], [29]. This process, known as EMT, has been described during the progression of different disease conditions such as cancer metastasis and fibrotic scarring in proliferative vitroretinopathy [21], [23]. During EMT, epithelial cells gradually lose their epithelial morphology and acquire mesenchymal gene expression profiles. Some of the EMT markers upregulated in transformed cells are glial fibrillary acidic protein (GFAP), vimentin, cyclin D1 (cD1), alpha smooth muscle actin (α-SMA), Snail1, C-terminal binding protein 1 (CtBP1) and N-Cadherin (with corresponding loss of E-cad) [22], [24], [26], [27], [28], [29]. To test whether the RPE cell morphological changes induced by downregulation of ZO-1 or overexpression of ZONAB was due to RPE cells undergoing EMT, we tested for the expression of EMT markers in RNA isolated by laser capture microdissection (LCM) from frozen tissues. Cryosections from eyes subretinally injected with either LNT.ZONAB or LNT.shZO-1 were obtained after 10 days of vector administration and approximately 100 RPE cells were collected from within the treated areas by LCM. RT-PCR was performed on mRNA extracts using different primers for EMT marker amplification. Figure 8 shows that GFAP, vimentin, N-cadherin, cD1 and Snail1 were increased by overexpression of ZONAB or downregulation of ZO-1. We did not observe overexpression of α-SMA or CtBP1 indicating that either these markers are not involved in early EMT, or α-SMA is not involved in ZONAB-mediated RPE transformation. We also used this approach to confirm the data obtained by inmunofluorescence and assess the levels of downregulation of ZO-1 or overexpression of ZONAB (Fig. 2 and 3). Thus, these results demonstrate that manipulating the expression of ZO-1 or ZONAB increases cell proliferation, alters epithelial phenotype and induces expression of five markers of EMT.

### RPE morphological disruption induced by manipulation of ZO-1 or ZONAB expression causes features of RPE dysfunction in fluorescein angiography

**Figure 9:**
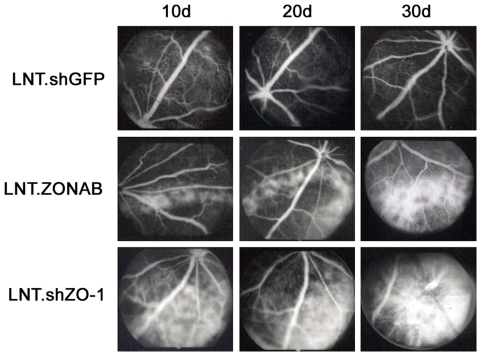
Downregulation of ZO-1 or overexpresion of ZONAB induce changes in fluorescein angiograms. Late-phase fluorescein angiograms of RNAi-treated eyes were obtained at 10, 20 and 30 days after subretinal injection of vectors (10^8^ T.U./ml). No abnormal changes in fluorescence were observed in LNT.shGFP injected eyes. Marked hyperfluorescence, indicating RPE cell loss, was seen in either LNT.shZO-1 or LNT.ZONAB treated eyes. Increasing intensity of the hyperfluorescence between timepoints suggests that RPE cell loss progressed over time. n = 4 per treatment group.

To evaluate the functional consequences of manipulation of ZO-1 or ZONAB expression in RPE cells, we next assessed whether the changes in the RPE morphology lead to functional alterations by fluorescein angiography. This technique demonstrates the integrity of the RPE tissue because a confluent RPE monolayer normally masks choroidal hyperfluorescence whereas loss of RPE function exposes it. Fluorescein angiography is used clinically to assess widespread loss of RPE cells in atrophic age-related macular degeneration, for example, where geographic RPE atrophy results in unmasking of the underlying choroidal hyperfluorescence. Figure 9 shows the results of fluorescein angiography at different time points (10, 20 and 30 days) following subretinal injections of LNT.ZONAB or LNT.shZO-1 at a titre of 10^8^ T.U./ml. In each case there was a progressive increase in fluorescence in the areas of the retina exposed to the vector. The speckled conformation of hyperfluorescence indicated discontinuities in the RPE with, at a latest time point (60 days p.i.), evidence of extensive RPE cell loss and retinal degeneration (data not shown). No fluorescein leakage was observed between early and late phase angiographs indicating that the integrity of the choroidal and retinal vasculature was not affected by subretinal delivery of the lentiviral vectors. Thus, manipulation of ZO-1 or ZONAB expression *in vivo* not only affects RPE morphology but also leads to a loss of at least some RPE functions.

## Discussion

Cell-cell adhesion is essential for the morphological integrity as well as the control of proliferation and differentiation of epithelial cells. Here, we demonstrate that alteration of the levels of the TJ components ZO-1 and ZONAB leads to changes in RPE cell proliferation, differentiation and function. Downregulation of ZO-1 or the overexpression of ZONAB led to the induction of cell proliferation and altered morphology correlating with expression of five EMT markers: GFAP, vimentin, N-cad, Snail1 and cD1. This study not only describes a novel approach to assess the role of TJ proteins in RPE function *in vivo* but also demonstrates that the two TJ-associated signalling proteins ZO-1 and ZONAB play a critical role in RPE homeostasis *in vivo*.

ZO-1 was the first TJ component to be identified [30]. ZO-1 deficiency in mice causes an embryonic lethal phenotype associated with defected yolk sac angiogenesis and apoptosis of embryonic cells [31]. In cells in two dimensional cultures, downregulation of ZO-1 has a mild effect on cell-cell junction assembly and a regulatory role in epithelial cell proliferation and gene expression [6]. However, in cells in three dimensional cultures, downregulation of ZO-1 affects epithelial morphogenesis [10]. ZO-1 has multiple protein-protein interaction domains [32]. The SH3 domain is necessary and sufficient to regulate cell proliferation and interacts with the Y-box transcription factor ZONAB [9]. The cytoplasmic sequestration of ZONAB by ZO-1 regulates its nuclear localisation and, hence, its effect on gene expression. The ZO-1/ZONAB pathway regulates cell cycle progression and epithelial morphogenesis in cells in culture [9], [10]. In this study we have demonstrated that downregulation of ZO-1 induced RPE proliferation and de-differentiation that eventually resulted in cell loss and retinal degeneration. Overexpression of ZONAB resulted in a very similar phenotype, suggesting that ZONAB activation is the main reason for the observed effects in response to depletion of ZO-1 expression in RPE cells. Downregulation of ZO-1 by LNT.shZO-1 has previously been shown to stimulate the transcriptional activity of ZONAB in epithelial cells in culture as ZO-1 functions as an inhibitor of ZONAB [9], [10]. In this study, reduced ZO-1 expression in RPE cells using the same vector also resulted in an increase in ZONAB staining. A possible explanation for this might be that depletion of ZO-1 led to activation of ZONAB, which might in turn have caused a positive feedback loop on its expression as ZONAB is known to be upregulated during proliferation [9], [11]. The similarities of the phenotypes observed following manipulation of ZO-1 or ZONAB levels together with their known biochemical and functional interactions, such as increase of cell proliferation and cyclin D1 expression, suggest that ZO-1 and ZONAB exert their effects on the RPE by, at least in part, a common molecular pathway. This pathway is likely to involve transcriptional activation of ZONAB. However, it might involve alternative mechanisms. For example, ZONAB is a Y-box factor which are also known to participate in cytoplasmic processes such as mRNA translation [33]. Such a possibility is further supported by the strong cytoplasmic staining of ZONAB in RPE cells.

Increase in ZONAB activity and ZO-1 downregulation resulted in an EMT-like phenotype. The fact that RPE cells have expression of RPE65 at similar time point that they start to upregulated EMT markers (Figure 8) suggest that 10 days is an earlier stage of the EMT-like phenotype. Nevertheless, de-differentiation seemed to occur more slowly than induction of proliferation, suggesting that cell cycle entry is a primary effect, whereas de-differentiation might be a consequence of such early changes. As the fraction of proliferative cells was relatively small, it could be that de-differentiation is triggered by these few cells via altered expression of adhesion proteins and/or secreted factors and that non-proliferative cells de-differentiate due to a bystander effect. However, it is also possible that morphological changes take longer to develop, but that yet to be identified early ZONAB targets do drive de-differentiation. It will thus be important to identify ZONAB target genes in RPE cells on a genome wide scale and design approaches to stimulate *in vivo* RPE proliferation independent of ZONAB to test whether induction of proliferation in even only a fraction of the cells is incompatible with the differentiated RPE phenotype.

RPE cells not only started to express mesenchymal markers, their cuboidal morphology changed to a flattened structure lacking the clear morphological hallmarks of RPE cells such as clear cell junctions, apical microvilli and basal infoldings. Together with the induction of cell proliferation, loss of epithelial morphology and EMT marker expression, these are all features of proliferative vitreoretinopathy (PVR) [21], [19], [34], a condition of exaggerated peri-retinal gliois induced by retinal detachment that is believed to be caused by proliferation and transdifferentiation of RPE cells. RPE de-differentiation has been shown to occur both in vitro [28] and in vivo [21] upon EMT marker overexpression and loss of TJ signalling components in PVR [35], [36] whereas EMT marker expression has also been linked with AMD [37]. Furthermore, aberrant or reduced retinal ZO-1 expression has been associated with blood-retinal barrier breakdown in diabetic retinopathy [38], [39]. Thus, certain features of the induced phenotype are common to disorders of the human RPE.

In this study we have observed that an increase in ZONAB activity results in increased RPE cell proliferation and altered RPE morphology, suggesting RPE dysfunction. As the postnatal RPE is no longer proliferative [19], the observed phenotype suggests that postnatally increased RPE proliferation could lead to RPE dysfunction and EMT. Furthermore, RPE dysfunction can lead to retinal degeneration in different animal models [16]. Alternatively, epithelial transformation could be one possible reaction of RPE cells in response to induced cellular stress caused by loss of contact inhibition due to retinal detachment or injury. ZONAB activation has also previously been linked to the cellular stress response [40]. It is therefore possible that the phenotype we observed might also result from a cellular stress response in the RPE. It will thus be interesting to test if Apg-2, which is responsible for ZONAB activation in response to heat shock, is also important for RPE homeostasis and, if so, what types of retinal stress conditions stimulate Apg-2 to activate ZONAB in the RPE. Nevertheless, the molecular function of ZONAB in healthy RPE is currently not known.

In this study, we demonstrated efficient lentivirally-mediated RNA interference and expression of junctional proteins in RPE cells *in vivo* that can be used to study the role of cell-cell adhesion-associated signalling mechanisms in a mature epithelial tissue. The induced phenotypes highlight the importance of ZONAB and ZO-1 in RPE homeostasis *in vivo*. However, additional characterisation of ZONAB involvement in EMT and cellular stress responses are required to define the underlying molecular mechanism. Furthermore, analysis of ZO-1 and ZONAB in human tissues derived from patients with different retinopathies will help to elucidate the functional contributions of this TJ-associated signalling pathway in retinal physiology and pathology.
