# Correction: The Tight Junction Associated Signalling Proteins ZO-1 and ZONAB Regulate Retinal Pigment Epithelium Homeostasis in Mice

**DOI:** 10.1371/journal.pone.0295782

**Published:** 2023-12-07

**Authors:** Anastasios Georgiadis, Marion Tschernutter, James W. B. Bainbridge, Kamaljit S. Balaggan, Freya Mowat, Emma L. West, Peter M. G. Munro, Adrian J. Thrasher, Karl Matter, Maria S. Balda, Robin R. Ali

The sample in Fig 5A [1] was inadvertently used to represent the Fig 5D results. The panel in Fig 5D has been replaced with the correct image. Original microscopy images for Fig 5 are provided in S1 File.

Additionally, the ×20 magnification image for Fig 5A [1] does not match the corresponding ×40 magnification image. The ×20 magnification image in Fig 5A has therefore been replaced.

The primary data underlying the rest of the results reported in this article are no longer available.

**Fig 5:**
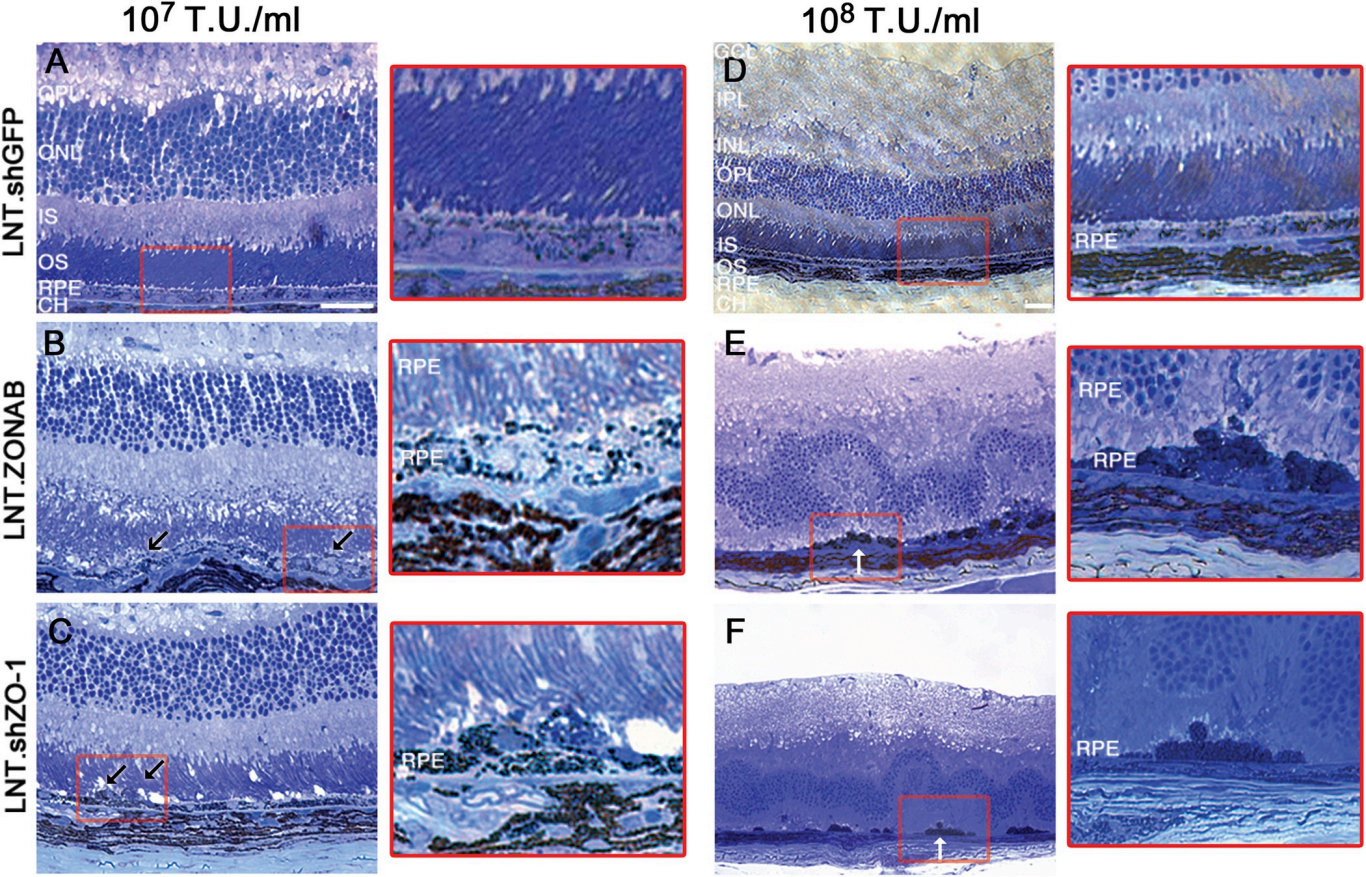
Downregulation of ZO-1 or overexpression of ZONAB affects retinal morphology. Retinal semithin sections were obtained after 10 days of subretinal injection of LNT.shGFP (**A,D**), LNT.ZONAB (**B,E**) or LNT.shZO-1 (**C,F**) at 10^7^ T.U./ml (left panel, ×40 magnification) and 10^8^ T.U./ml (right panel, ×20 magnification). Areas in red rectangles are shown in higher magnification. The LNT.shGFP treated eyes (**A,D**) exhibit normal retinal architecture showing that there are no adverse effects induced either by the lentiviral vector itself or by the expression of shRNA. Following injection of either LNT.ZONAB (**B,E**) or LNT.shZO-1 (**C,F**) signs of RPE puknosis and multilayerisation (black arrows) as well as retinal folding and rosette formation in areas corresponding to those with severe RPE abnormalities (white arrows) were observed. GCL, ganglion cell layer. IPL, inner plexiform layer. INL, inner nuclear layer. OPL, outer plexiform layer. ONL, outer nuclear layer. IS, inner segments. OS, outer segments. RPE, retinal pigment epithelium. CH, choroid. Size bar, 20 µm. n  =  4 per treatment group.

## Supporting information

S1 File Original images underlying Fig 5. (ZIP)
