# Supplementary figures and images for: Correction: The Tight Junction Associated Signalling Proteins ZO-1 and ZONAB Regulate Retinal Pigment Epithelium Homeostasis in Mice
Source: PLoS One. 2023 Dec 7;18(12):e0295782. doi: 10.1371/journal.pone.0295782 (PMC10703191; doi:10.1371/journal.pone.0295782)

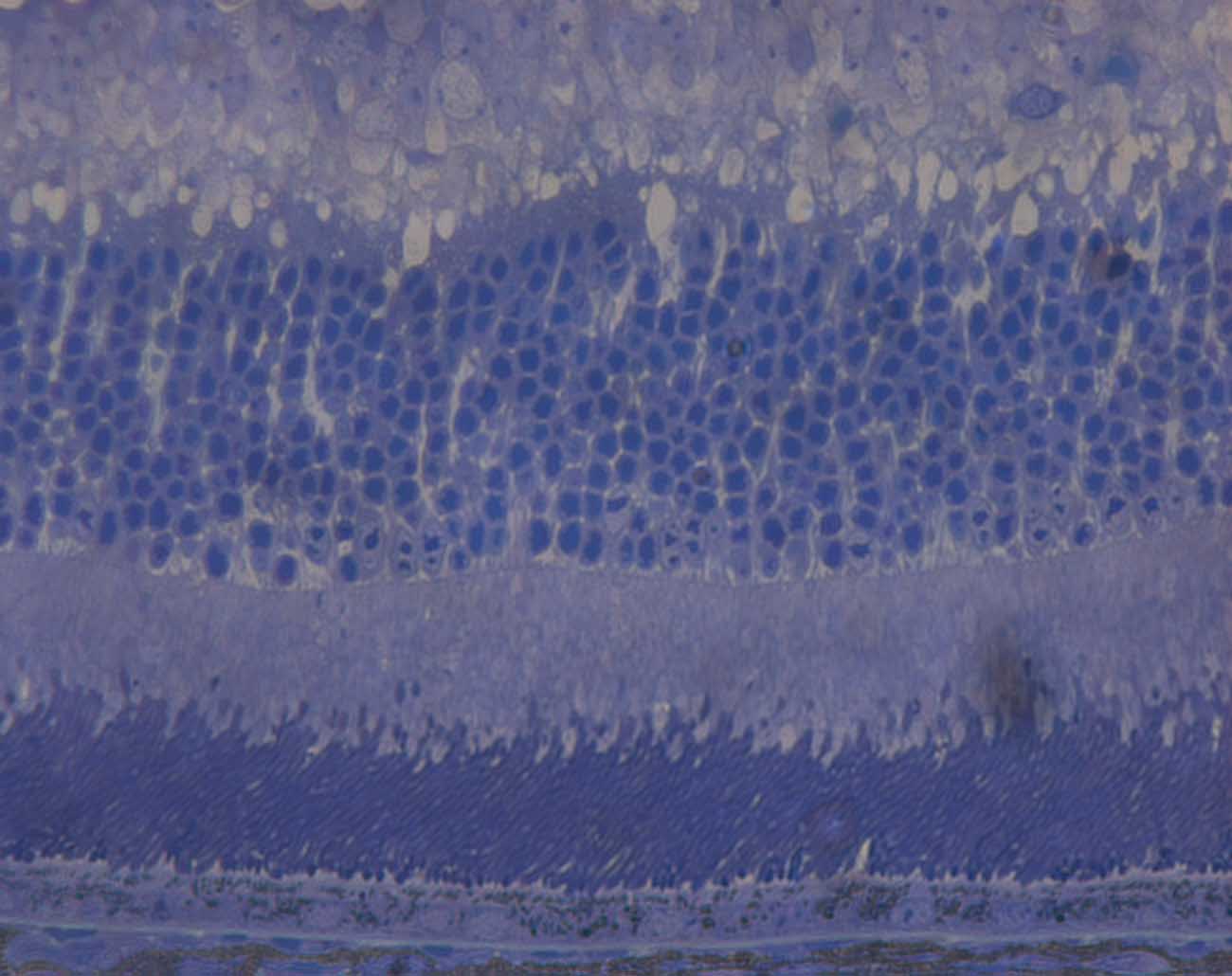

Supplement: S1 File — (ZIP) [file pone.0295782.s001.zip › S1 File/Fig5A shGFP 1e7.jpg]

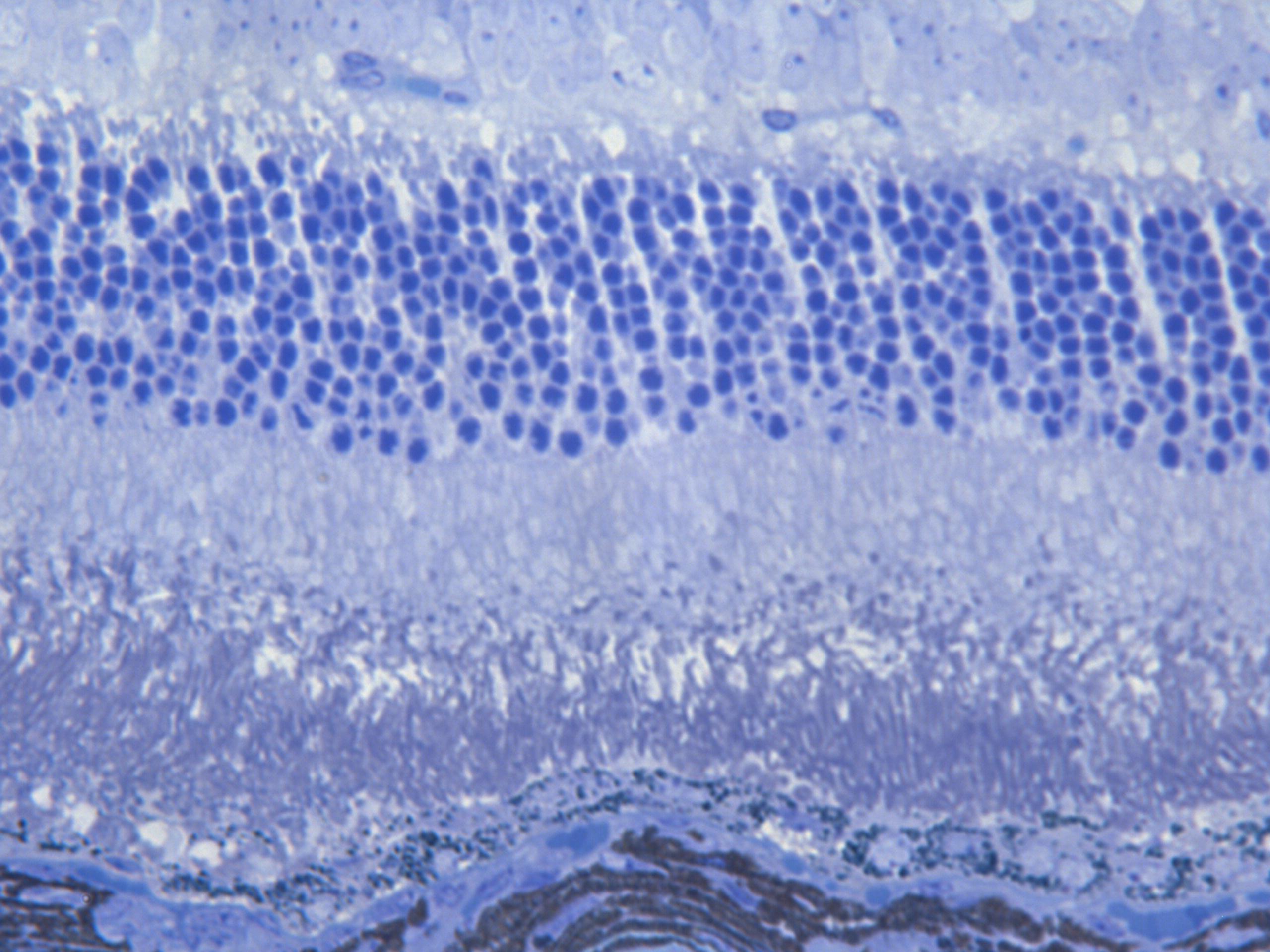

Supplement: S1 File — (ZIP) [file pone.0295782.s001.zip › S1 File/Fig5B ZONAB 1e7.jpg]

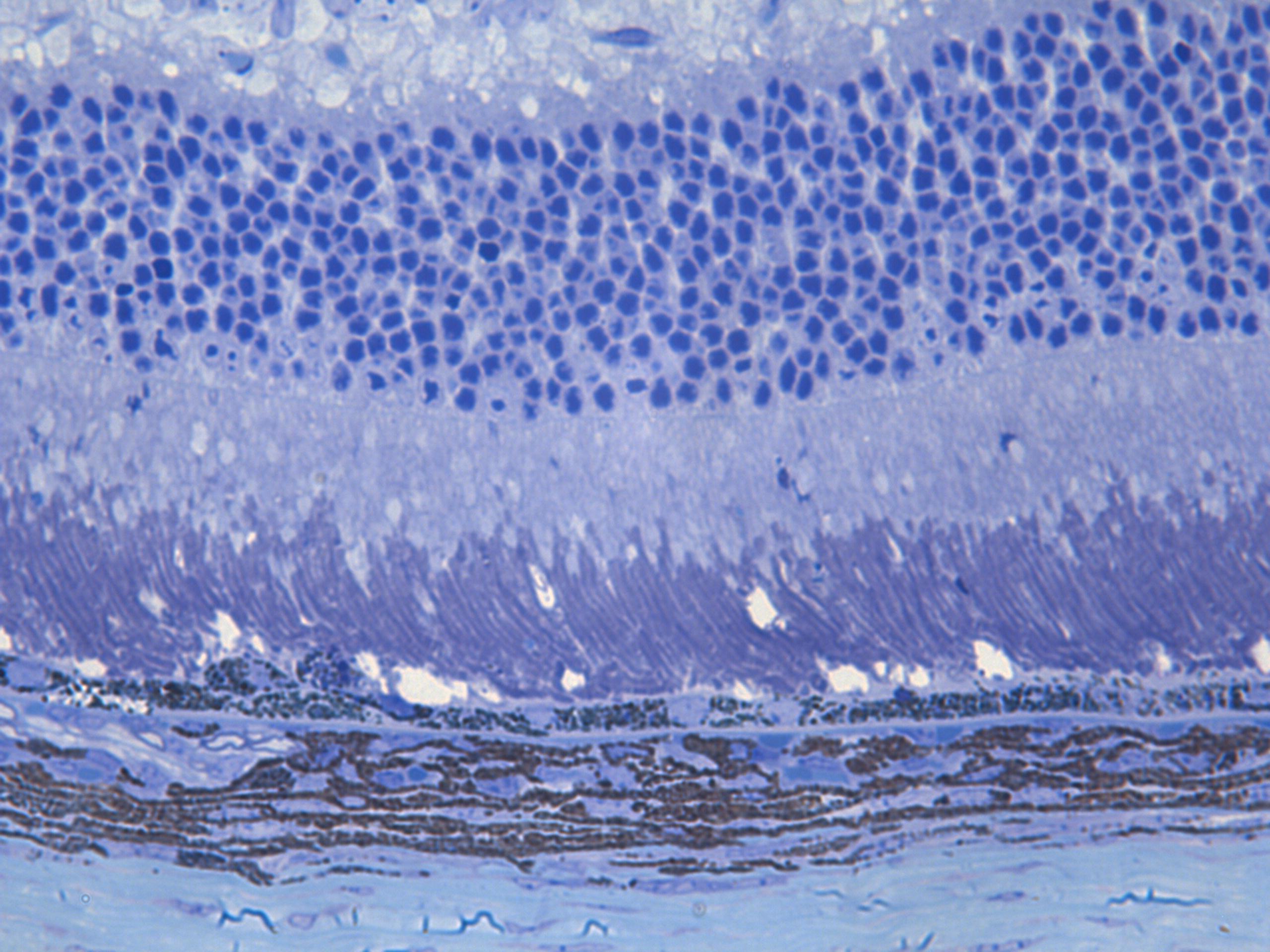

Supplement: S1 File — (ZIP) [file pone.0295782.s001.zip › S1 File/Fig5C shZO1 1e7.jpg]

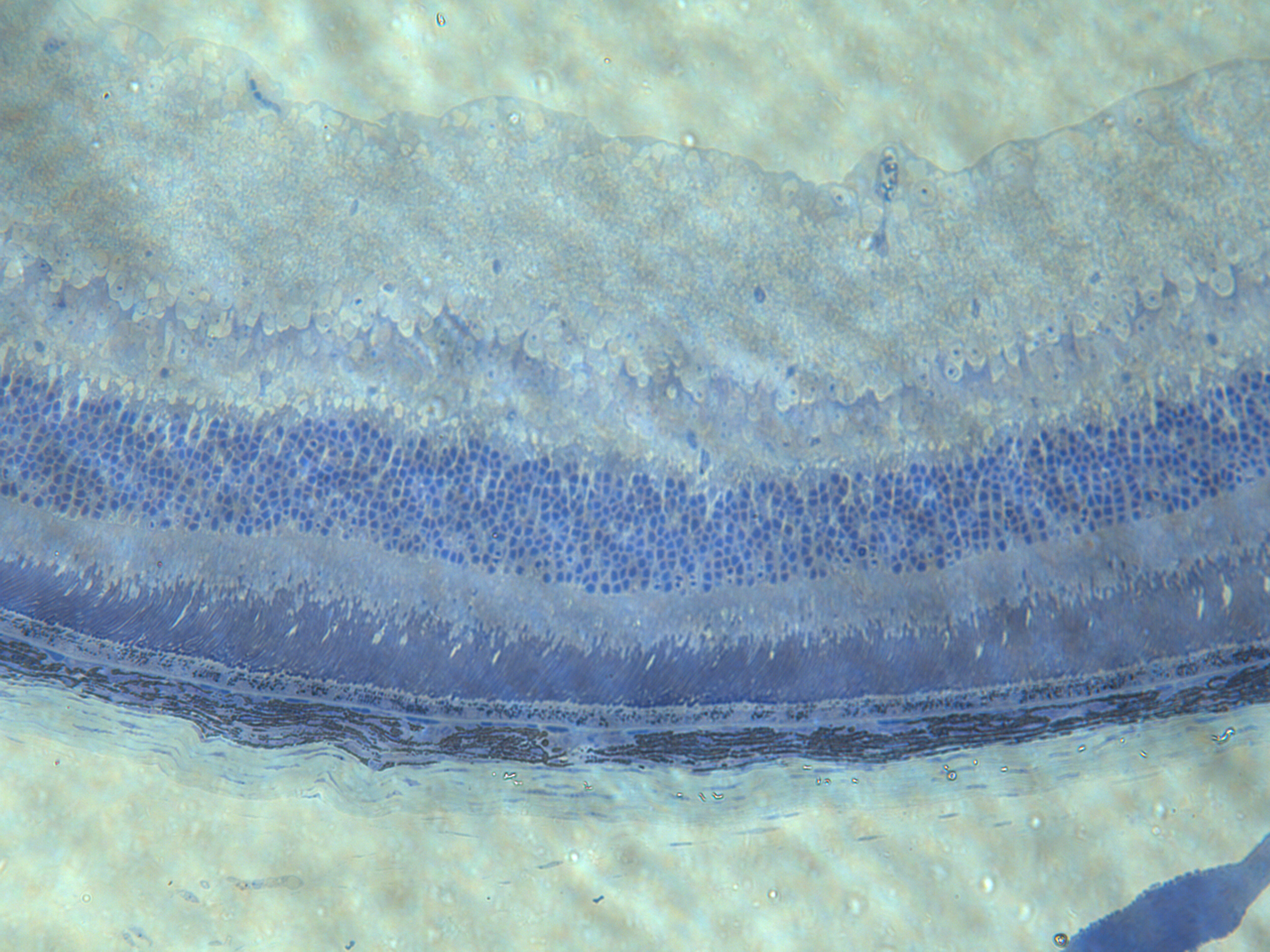

Supplement: S1 File — (ZIP) [file pone.0295782.s001.zip › S1 File/Fig5D shGFP 1e8.tif]

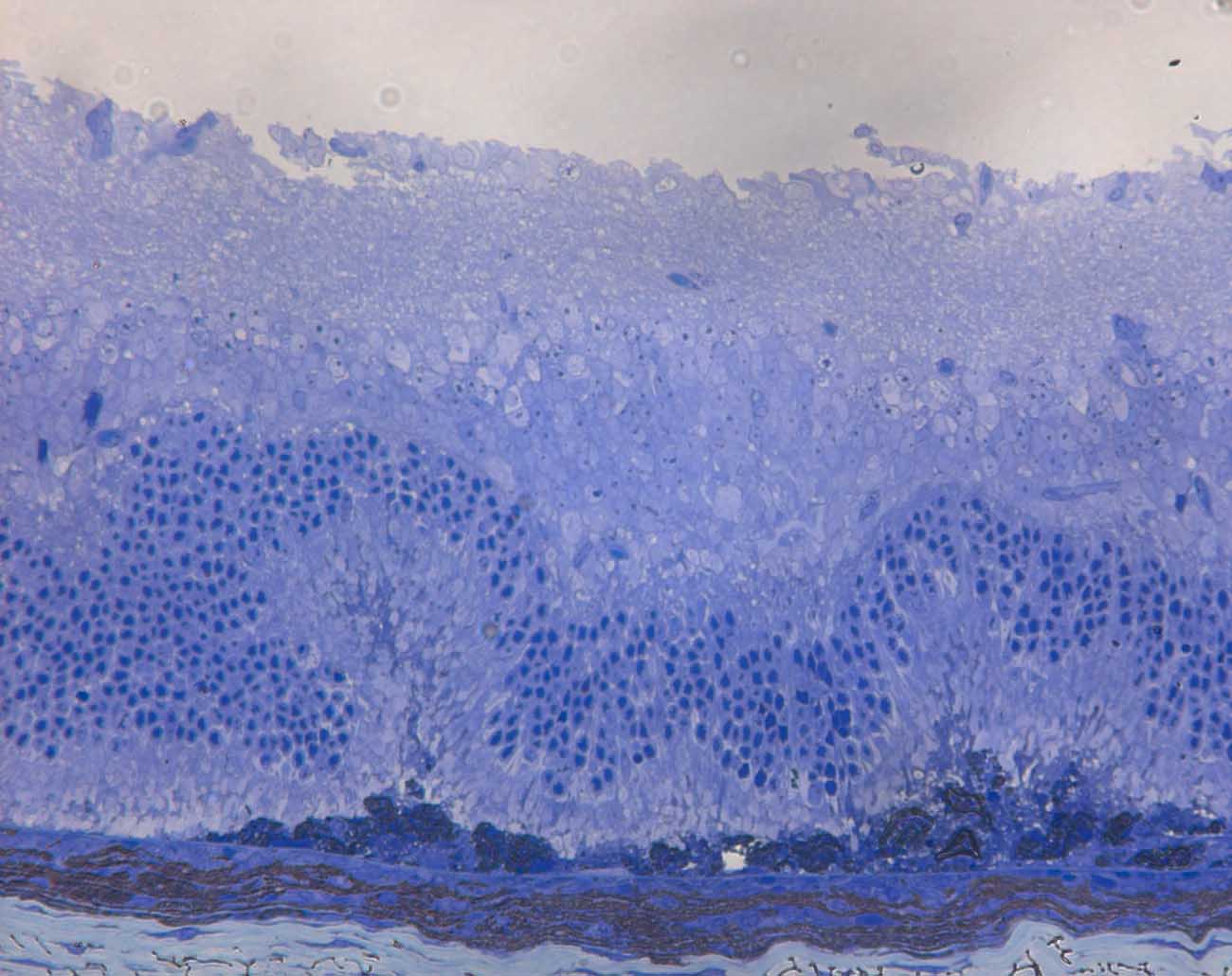

Supplement: S1 File — (ZIP) [file pone.0295782.s001.zip › S1 File/Fig5E ZONAB 1e8.jpg]

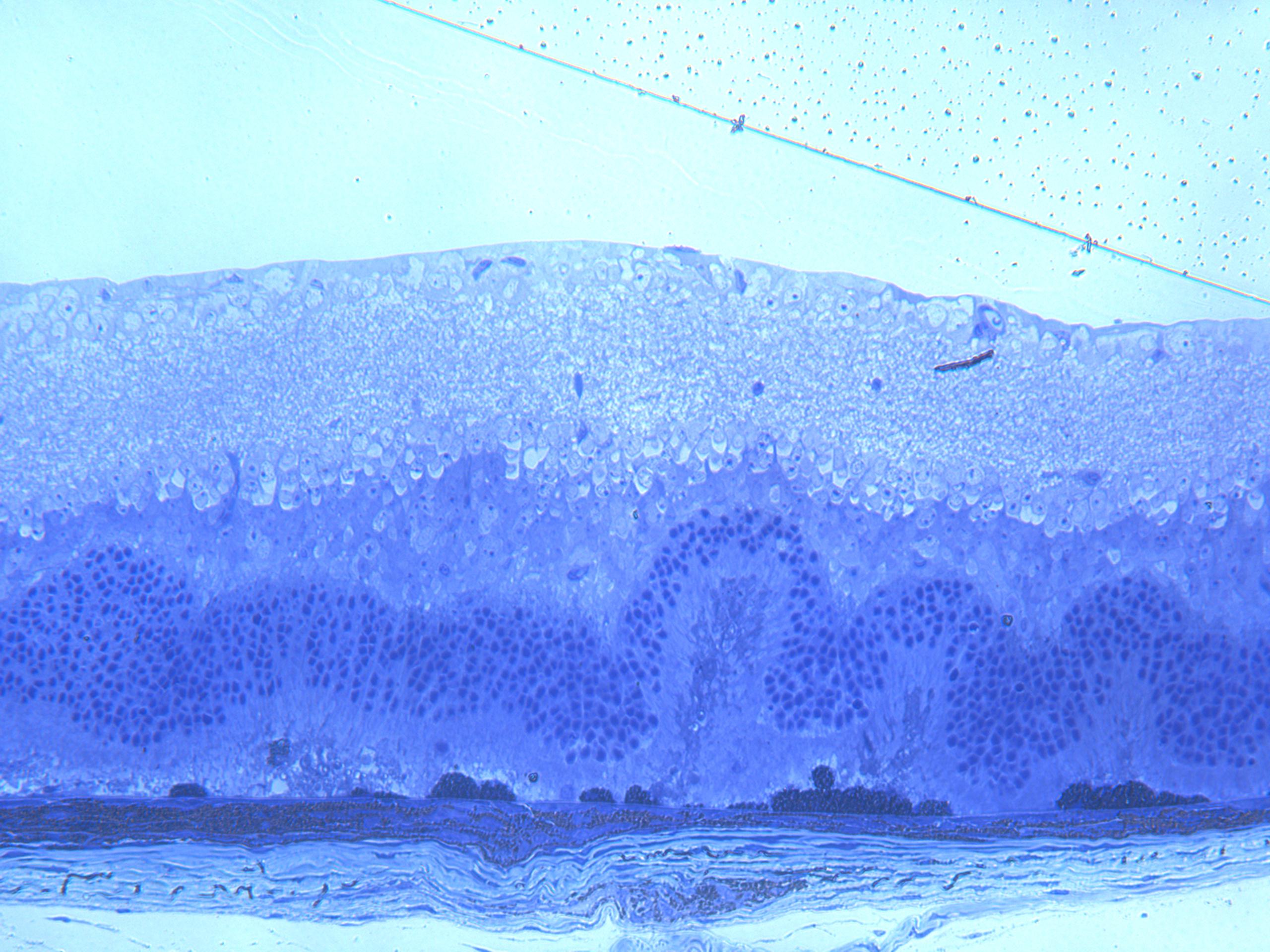

Supplement: S1 File — (ZIP) [file pone.0295782.s001.zip › S1 File/Fig5F shZO1 1e8.jpg]
